# Performance Characterization of Hot Mix Asphalt with High RAP Content and Basalt Fiber

**DOI:** 10.3390/ma13143145

**Published:** 2020-07-15

**Authors:** Zhengguang Wu, Chen Zhang, Peng Xiao, Bo Li, Aihong Kang

**Affiliations:** 1 College of Civil Science and Engineering, Yangzhou University, Yangzhou 225127, China; zgwu@yzu.edu.cn (Z.W.); Zhangchenyzu@126.com (C.Z.); libo@yzu.edu.cn (B.L.); ahkang@yzu.edu.cn (A.K.); 2 Research Center for Basalt Fiber Composite Construction Materials, Yangzhou University, Yangzhou 225127, China

**Keywords:** asphalt mixtures, RAP, basalt fiber, rutting, cracking, moisture susceptibility

## Abstract

Incorporating reclaimed asphalt pavement (RAP) into asphalt mixtures achieves astonishingly environmental and economic benefits. However, there is hesitation to use higher RAP content due to the concern regarding the deterioration in pavement performance, especially the cracking resistance. Basalt fiber has been considered an effective additive to reinforce the performance of asphalt mixtures and, subsequently, the reinforcement effect is also expected for high-RAP content mixtures. Therefore, this study investigated the effect of basalt fiber on the pavement performance of asphalt mixtures with 0%, 30%, 40%, and 50% RAP contents against high-temperature performance, moisture susceptibility, low-temperature and intermediate-temperature cracking resistance, based on the wheel-tracking test, the uniaxial penetration test, the freeze-thaw splitting test, the low-temperature bending beam test, the semicircular bend fracture test and the indirect tensile asphalt cracking test, respectively. In addition, a performance-space diagram was developed to determine the mixture performance shift caused by basalt fiber. The results showed that adding basalt fiber compensated for the detrimental effect caused by RAP, leading to significant enhancement in moisture susceptibility and low- and intermediate-temperature cracking resistance of mixtures with high RAP content, along with the enhancement in high-temperature performance, indicating that basalt fiber can contribute to the use of high RAP content.

## 1. Introduction

The application of reclaimed asphalt pavement (RAP) in asphalt mixtures has been vigorously encouraged and promoted over the past decades due to the desirable environmental benefit, along with the cost reduction [1,2,3,4]. The use of RAP has been promoted from low-value use (as unbound layer materials [5], etc.) to high-value use (as surface materials, etc.), and became commonplace in some developed countries. It is reported that over 99% of RAP is recycled as alternative road materials in the USA [6], while the RAP use rate is more than 80% in Europe [1]. Chinese transport agencies are making great efforts to achieve a RAP use rate of over 80% by the end of 2020 [7]. However, the RAP dosage used in asphalt mixtures is still surprisingly low. By weight of total mixture, the RAP dosage is still around 20% in most states in the USA [6]. Meanwhile, RAP is not even commonly used in the surface layer of asphalt pavement in China. 

The major reason for the hesitation to use high RAP content in asphalt mixtures (especially surface layer) is that excessive RAP would make asphalt mixtures stiffer and more brittle [8,9], leading to potential deterioration in cracking resistance. The Federal Highway Administration (FHWA) considers that mixtures with more than 25% RAP can be recognized as high-RAP content mixtures [10], since the performance of asphalt mixtures changed dramatically when RAP content reached 30%, even compared to those with 20% RAP [11]. Tremendous studies have been conducted to investigate the effect of RAP on the performance of asphalt mixtures, especially on cracking resistance. Zhou’s research [12] pointed out that the use of RAP increased the cumulative rate of fatigue damage, subsequently resulting in shortened fatigue life of asphalt mixtures, which has been proved by some other investigations [13,14]. The use of RAP also further diminished the low-temperature property of asphalt mixtures, and commonly made the mixtures prone to thermal cracking [9,15], especially in cold regions. Winkle’s investigation results from laboratory and field evaluation showed that with an increasing RAP content, the stiffness of asphalt mixtures increased, while fracture energy decreased [16]. Besides, adding RAP also impacted negatively on the short-term and long-term aging behaviors of asphalt mixtures [17], in addition to the self-healing property [18].

The commonly used method to compensate for the adverse effect of RAP is to introduce rejuvenators or softer asphalt binders into asphalt mixtures. A rejuvenator or softer asphalt binder can adjust the compositions of the aged asphalt binder in order to recover its rheological properties to some degree [19,20]. Nevertheless, rejuvenated asphalt binders present inferior re-aging resistance than fresh binders, despite the desirable performance at an early stage [21,22]. As a result, the long-term instability and accelerated performance deterioration have been considered to be the main deficiencies of rejuvenated asphalt mixtures [23,24,25].

Another solution is to incorporate fibers with RAP into asphalt mixtures, as fibers have been proved to be effective reinforcing additives for asphalt mixtures [26]. The typical types of fibers used for asphalt mixtures include: mineral fibers (glass fiber, etc.), polymer fibers [27] (polyolefin–aramid, polyacrylonitrile, etc.), cellulose fiber, carbon fiber and steel fiber [28], along with a few new fibers currently being tested, such as aminated graphene fiber [29], kenaf and goat wool [30], etc. The results showed that incorporating fibers with RAP could effectively improve the tensile strength, rutting resistance and moisture susceptibility of asphalt mixtures, in addition to enhancing the resistance to crack initiation and propagation significantly [31,32,33]. However, each type of fiber presents its own disadvantages when used in asphalt mixtures. For instance, cellulose fiber mainly has the function of absorbing and stabilizing asphalt binders rather than enhancing the strength of asphalt mixtures [34]. Polymer fibers exhibit inferior durability and dispersibility in asphalt mixtures [35], while glass fiber shows poor adhesion with asphalt binder due to its smooth texture [36].

Using basalt fiber into asphalt mixtures has gained increasing attention over the past years. Basalt fiber is made from molten basalt rocks, and is considered as an innovative type of cost-effective and environmentally friendly mineral fiber. Basalt fiber possesses comparable properties with carbon fiber T300, such as, high fracture strength, excellent acid and alkali resistance, superior high-temperature and low-temperature resistance, etc. [37]. A few studies have been dedicated to the effect of basalt fiber on the performance of asphalt mixtures, and the results confirmed the desired enhancing effect. The mixtures with basalt fiber showed superior resistance to high-temperature permanent deformation, low-temperature and intermediate-temperature cracking, moisture damage, and fatigue failure [34,38,39,40,41,42]. Besides, asphalt mixtures with basalt fiber presented better mechanical performance and drain-down resistance when compared to those mixed with glass fiber [41,43]. Based on the above analysis, adding basalt fiber has anticipated potentiality to improve the performance of high-RAP content mixtures, especially cracking resistance. However, very limited amount of research has been conducted on this topic.

Therefore, the primary objective of this study was to investigate the performance of hot mix asphalt with high RAP content and basalt fiber. To achieve the goals, asphalt mixture samples containing 0%, 30%, 40% and 50% RAP reinforced with basalt fiber were fabricated. Then, the high-temperature deformation performance, moisture susceptibility, low-temperature and intermediate-temperature cracking resistance of all the samples were evaluated. The results were also compared to those of the control mixtures without basalt fiber in order to better understand the reinforcing effect caused by basalt fiber.

## 2. Materials and Sample Fabrication

### 2.1. Raw Materials

#### 2.1.1. Basalt Fiber

**Figure 1:**
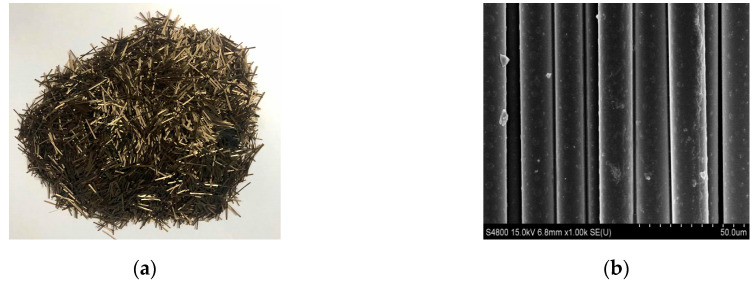
Morphologies of basalt fiber: (**a**) appearance; (**b**) scanning electronic microscope image.

A type of high-grade basalt fiber was selected as the reinforcing additive for this study. The morphologies of the fiber are illustrated in Figure 1. The main physical and mechanical properties are summarized in Table 1. It can be seen from Figure 1 that basalt fiber is golden brown in color, with a regular cylindrical shape on the micron scale.

#### 2.1.2. Asphalt Binder and New Aggregates

A type of styrene-butadiene-styrene (SBS) modified asphalt binder was used in this study. The properties of the binder are listed in Table 2. Limestone coarse and fine aggregates were selected, with the density of 2.698 g/cm^3^ and 2.651 g/cm^3^, respectively. Limestone powders with a density of 2.714 g/cm^3^ were chosen as mineral fillers.

#### 2.1.3. RAP Material

The RAP material was obtained from the surface layer of S356 freeway in Jiangsu Province, China. The asphalt binder was extracted according to JTG E20, and the binder content, binder properties and RAP gradation were tested. Four replicates were conducted for each test. Table 3 summarizes the binder content and properties of the extracted binder. Kang [44] proposed a method to determine the aging degree of extracted binder from RAP, in which the extracted binder can be classified into six aging grades based on the penetration and viscosity values, as shown in Table 4. According to Kang’s method, the extracted binder was considered to be grade II—mild aging.

### 2.2. Sample Fabrication

#### 2.2.1. Mixture Gradation Design

The dense-graded gradation of asphalt concrete called AC-13 with the nominal maximum aggregate size (NMAS) of 13.2 mm was selected for this study. Three RAP contents, namely, 30%, 40%, and 50% were chosen to fabricate the asphalt mixtures. In addition, another set of asphalt mixtures were reinforced by basalt fiber. According to the newly issued guideline “Technical Guideline for Construction of Asphalt Pavement with Basalt Fiber” (T/CHTS 10016-2019), basalt fibers with a length of 6 mm and the dosage of 0.3% by total weight of the mixture were used. The mixture gradations are illustrated in Table 5. It can be seen that the gradations of the mixtures with 30%, 40%, and 50% RAP were nearly the same. The optimum asphalt–aggregate ratio of each mixture was determined by the Marshall design method, which was 5.00%, 4.58%, 4.47% and 4.34% for the mixture with 0%, 30%, 40%, and 50% RAP, respectively. Meanwhile, when basalt fiber was added, the optimum asphalt-aggregate ratio of each mixture was determined as 5.20%, 4.78%, 4.67% and 4.54%, respectively.

#### 2.2.2. Mixing and Compaction

The mixing process of the high-RAP content mixture was in accordance with the Chinese standard JTGE20 T0702. In terms of the mixtures with basalt fiber, a “dry mixing process” was essential in order to make the fibers disperse as homogeneously as possible, which meant that basalt fiber should be mixed with the new aggregates for 90 s firstly, and then mixed with RAP for another 90 s before adding asphalt. Regarding the compaction process, there was no obvious impact of basalt fiber on the compaction work. However, it is worth noting that the asphalt mixture samples with basalt fiber, which was compacted by a superpave gyratory compactor, should be demolded at relatively low temperatures (<80 °C), as samples were more prone to collapse when compared to the control samples without basalt fiber. 

## 3. Experimental Methods

### 3.1. Wheel-Tracking Test

The wheel-tracking test was performed in accordance with the Chinese standard JTG E20 T0719 in order to evaluate the permanent deformation resistance of asphalt mixtures. Slab samples with the size of 300 mm × 300 mm × 50 mm were used for this test. The wheel speed was set to be 42 passes per minute with a default pressure of 0.7 MPa and test temperature of 60 °C. Based on the recorded rutting depth, dynamic stability (DS) was calculated by Equation (1). Generally, a higher DS value will be expected to achieve superior rutting resistance.

(1) $$DS=\frac{(t_{2}-t_{1})\times N}{d_{2}-d_{1}}\times C_{1}\times C_{2}$$

where $d_{1}$ is the rut depth at the timing of 45 min (mm), $d_{2}$ is the rut depth at the timing of 60 min (mm), $C_{1}$ and $C_{2}$ are experimental coefficients, $C_{1}$ = $C_{2}$ = 1.0 in this study, N means the numbers of wheel passing in one minute, N = 42 passes/min.

### 3.2. Uniaxial Penetration Test

**Figure 2:**
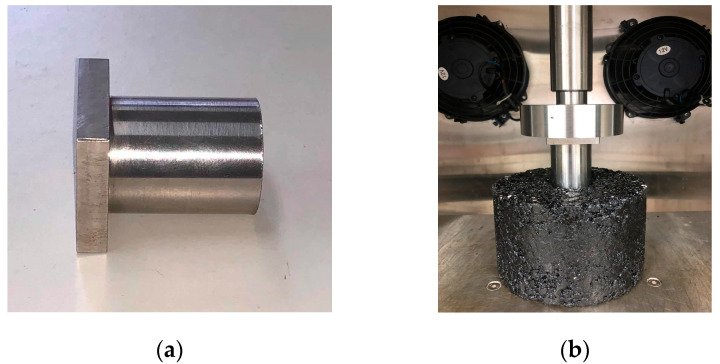
Images of the uniaxial penetration test: (**a**) plunger; (**b**) experimental set-up.

The uniaxial penetration test was employed in accordance with the Appendix F of the Chinese standard JTG D50 in to evaluate the high-temperature shear resistance of asphalt mixtures, as shown in Figure 2. Cylindrical specimens with a diameter of 150 mm and height of 100 mm were used. This test was also conducted at 60 °C, and the load was applied through a metal plunger (Figure 2a) with a loading rate of 1 mm/min. Procedure details of this test are available in the reference [45]. The shear strength can be expressed by Equation (2).

(2) $$\tau_{0}=f\times F/A_{c}$$

where $\tau_{0}$ is the shear strength (MPa), F is the maximum load (N), $A_{c}$ is the cross-section area (mm^2^), f = 0.350, representing the sample dimension correction coefficient.

### 3.3. Freeze-Thaw Splitting Test

The freeze-thaw splitting test was conducted to access the moisture susceptibility of asphalt mixtures, according to the Chinese standard JTG E20 T0729. Two groups of Marshall samples were used in this test. The unconditioned group samples were soaked in a water bath of 25 °C for 2 h, while the conditioned group samples were firstly frozen at −18 °C for 16h, then thawed in a water bath of 60 °C for 24 h, and finally soaked in a water bath of 25 °C for 2h before the splitting procedure. The indirect tensile strength ratio (ITSR) was defined to evaluate the strength loss caused by freeze-thaw conditions, as shown in Equation (3).

(3) $$ITSR=\frac{ITS\;of\;conditioned\;samples}{ITS\;of\;unconditioned\;samples}\times 100\%$$

### 3.4. Low-Temperature Bending Beam Test

The low-temperature bending beam test was carried out in accordance with the Chinese standard JTG E20 T0715 in order to evaluate the tensile property of asphalt mixtures at a low temperature. The dimension of the beam sample was 250 mm × 30 mm × 35 mm. This test was run at −10 °C with a loading rate of 50 mm/min by a three-point bending mode. The flexural-tensile strength, maximum flexural-tensile strain at failure point, and flexural stiffness modulus were calculated by Equations (4)–(6).

(4) $$R_{B}=3LP_{B}/2bh^{2}$$

(5) $$\varepsilon_{B}=6hl/L^{2}$$

(6) $$S_{B}=R_{B}/\varepsilon_{B}$$

where $R_{B}$ is the flexural-tensile strength (MPa), $P_{B}$ is the peak load at failure (kN), *ε_B_* is the flexural-tensile strain (με), $S_{B}$ is the flexural stiffness modulus (MPa), l is the mid-span deflection at failure (mm), L, b and h are the length, width and height of the beam sample (mm), respectively.

### 3.5. Semicircular Bend Fracture Test

The semicircular bend (SCB) fracture test was employed to determine the fracture potential of asphalt mixtures at intermediate temperature, according to the American standard AASHTO TP 124. The dimension of the SCB samples was 150 ± 1.0 mm in diameter, 50 ± 1.0 mm in thickness, and with a notch of 15 ± 1.0 mm. This test was conducted at 25 °C with a loading rate of 50 mm/min. The flexibility index (FI), which is rather sensitive to recycled materials, has been proposed to identify brittle mixtures that are prone to premature cracking. The FI was defined as the total fracture energy divided by the absolute value of the slope at the inflection point, as shown in Equations (7) and (8).

(7) $$G_{f}=\frac{W_{f}}{Area_{lig}}\times 10^{6}$$

(8) $$FI=\frac{G_{f}}{\left|m\right|}$$

where $G_{f}$ is the fracture energy (J/m^2^), $W_{f}$ is the work of the fracture (J), $Area_{lig}$ is the ligament area (mm^2^), |m| is the absolute value of the slope m at the inflection point (kN/mm).

### 3.6. Indirect Tensile Asphalt Cracking Test

**Figure 3:**
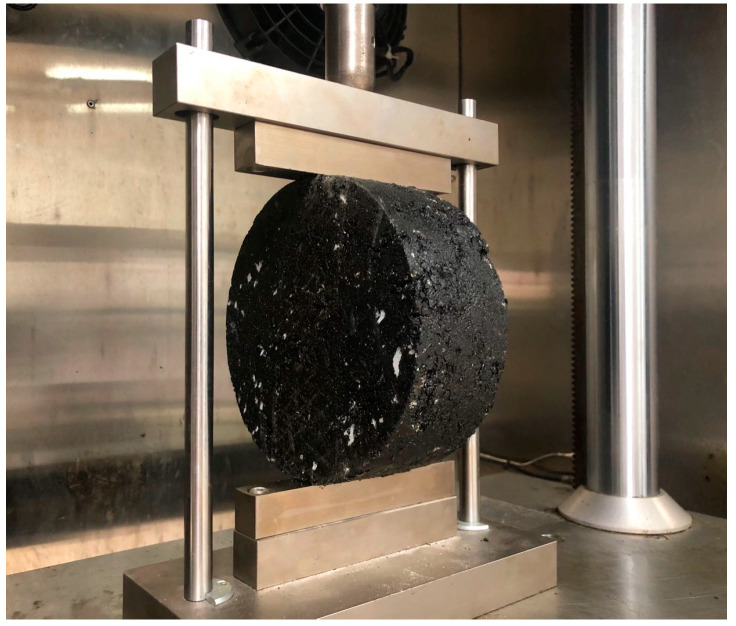
Image of the indirect tensile asphalt cracking test (IDEAL-CT).

**Figure 4:**
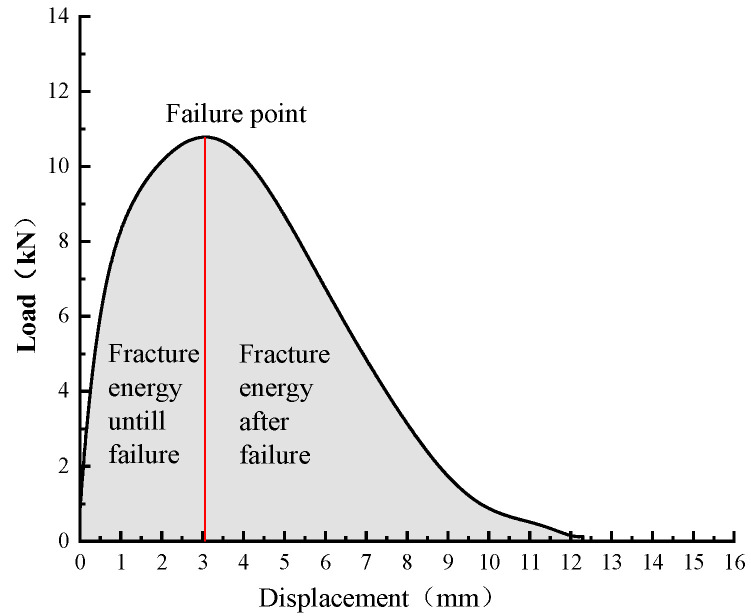
Illustration of fracture energy until failure.

The indirect tensile asphalt cracking test (IDEAL-CT) was carried out to evaluate the cracking resistance of asphalt mixtures with RAP. The IDEAL-CT, which was developed by Zhou [46], was conducted by similar procedures with the conventional indirect tensile strength test, as shown in Figure 3. The cylindrical specimens with a diameter of 150 mm and a thickness of 62 mm were used in this study. The air voids of all samples were kept within 7 ± 0.5%. This test was also run at 25 °C with a loading rate of 50 mm/min. CT_index_ was proposed as a simple and fast index to determine the crack growth rate of asphalt mixtures, which is defined by Equation (9). Fracture energy until failure was defined to determine the energy causing initial cracking, which is the area under the curve before the failure point, as shown in Figure 4.

(9) $$CT_{index}=\frac{G_{f}}{\left|m_{75}\right|}\times \frac{l_{75}}{D}$$

where $G_{f}$ is the fracture energy (J/m^2^), $\left|m_{75}\right|$ is the absolute value of the slope at the 75% inflection point of the peak load (kN/mm), $l_{75}$ is the displacement at the 75% point of the peak load (mm), and D is the diameter of the sample (mm).

Besides, three to six replicates were conducted for each test according to the corresponding requirement. The mean values of the replicates were resultantly used for analysis and discursion. In addition, error bars, which represent plus and minus one standard deviation, are also illustrated in the relevant figures.

## 4. Results and Discussion

### 4.1. Effect of Basalt Fiber on High-Temperature Performance of Asphalt Mixtures with RAP

#### 4.1.1. Wheel-Tracking Test Results

**Figure 5:**
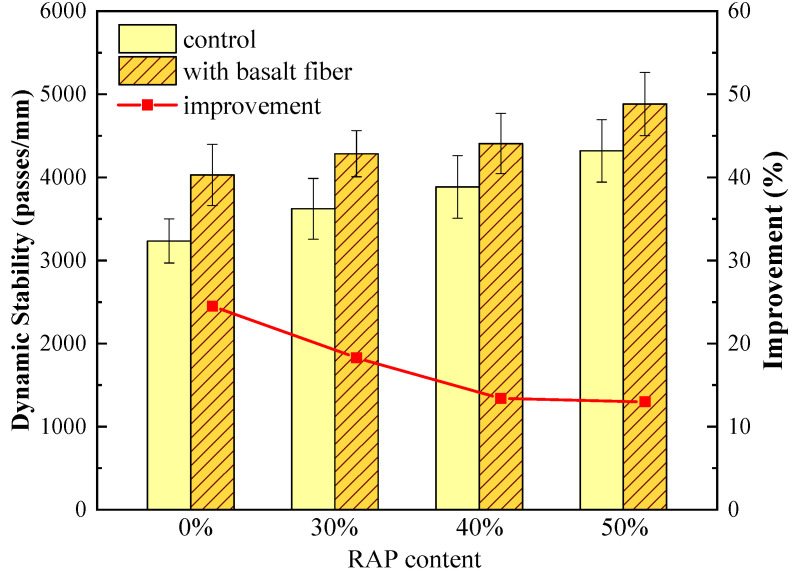
Wheel-tracking test results of control and basalt fiber-reinforced asphalt mixtures.

The dynamic stability results from the wheel-tracking test are illustrated in Figure 5. In terms of the control group samples, the dynamic stability values presented an increasing trend with an increasing RAP content. For instance, the dynamic stability of asphalt mixtures increased from 3235 passes/mm to 4320 passes/mm when RAP content increased from 0% to 50%. Higher dynamic stability means superior high-temperature deformation resistance. It infers that higher RAP content can result in a property that allows better resistance to high-temperature deformation for asphalt mixtures due to its stiffening impact.

With regard to the samples with a certain RAP content, the dynamic stability values of asphalt mixtures escalated significantly when basalt fiber was introduced, which was in accordance with the results from the reference [42]. The improvement ratio of dynamic stability caused by basalt fiber was calculated and illustrated as the red line in Figure 5. Compared to the control samples without basalt fiber, the dynamic stability values of fiber-reinforced asphalt mixtures with 0%, 30%, 40%, and 50% RAP increased by 24.5%, 18.3%, 13.4% and 13.0%, respectively. This indicates that the addition of basalt fiber can enhance the high-temperature deformation resistance of asphalt mixture to a great extent. However, this enhancing effect would be weakened by RAP content. The reason might be that basalt fiber can absorb some parts of the light components of the asphalt binder [41], resulting in higher viscosity of the binder and subsequently better high-temperature performance. However, less light components could be absorbed by basalt fiber when high RAP content was utilized in asphalt mixtures, leading to a reduction in the enhancing effect.

#### 4.1.2. Uniaxial Penetration Test Results

**Figure 6:**
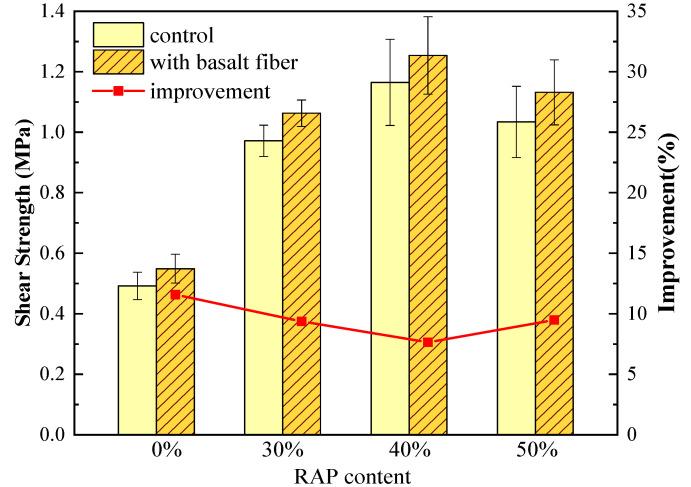
Uniaxial penetration test results of control and basalt fiber-reinforced asphalt mixtures.

The shear strength results from uniaxial penetration test are shown in Figure 6. As for the control group samples, it can be seen that asphalt mixtures exhibited a remarkable increase in shear strength when 30% RAP was added, reaching 97.6% higher than the asphalt mixtures with 0% RAP. It is worth noting that shear strength reached the maximum value when 40% RAP was used, and then declined a little bit by adding more RAP. These findings disagree with the dynamic stability results, which kept growing with increasing RAP content. This indicates that the stiff RAP material does significantly impact the high-temperature shear strength of asphalt mixtures, resulting in superior high-temperature performance. However, excessive RAP can not only cause cracking problems; it can also lead to a reduction in shear strength. Therefore, the uniaxial penetration test can provide a different perspective to evaluate the high-temperature performance, which may be used as a complementary approach to the wheel-tracking test.

In terms of the samples with basalt fiber, the shear strength values further increased compared to the control samples. The improvement ratio caused by basalt fiber was also plotted as the red line in Figure 6. Compared to the control samples without basalt fiber, the improvement ratio of fiber-reinforced asphalt mixtures with 0%, 30%, 40%, and 50% RAP increased by 11.6%, 9.4%, 7.6% and 9.5%, respectively. This indicates that the addition of basalt fiber can reinforce the shear strength of asphalt mixtures with RAP to some extent, but not as much as the effect caused by RAP.

### 4.2. Effect of Basalt Fiber on Moisture Susceptibility of Asphalt Mixtures with RAP

**Figure 7:**
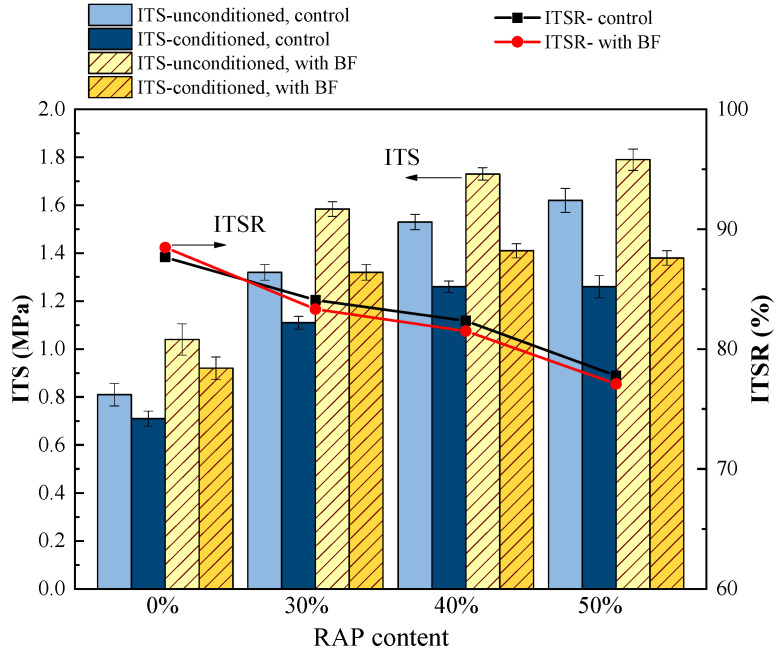
Freeze-thaw splitting test results of control and basalt fiber-reinforced asphalt mixtures.

The indirect tensile strength (ITS) and indirect tensile strength ratio (ITSR) results from the freeze-thaw splitting test are illustrated in Figure 7. With regard to the control group samples, it can be seen that asphalt mixtures presented a sharp fall in the ITSR by the increasing RAP content, as shown in the black line in Figure 7. The ITSR values declined from 87.7% to 77.8% when RAP content increased from 0% to 50%. Meanwhile, in terms of the samples with basalt fiber (BF), the ITSR values showed a similar decreasing trend to the control samples, as shown in the red line in Figure 7. Moreover, no distinct variations in the ITSR could be observed between the control and fiber-reinforced samples. It seemed that basalt fiber had no impact on the moisture susceptibility of asphalt mixtures with RAP. Nevertheless, it can be noticed from the bar charts in Figure 7 that the ITS values of the fiber-reinforced samples, for both unconditioned and conditioned ones, increased significantly compared to the control samples, indicating that the fiber-reinforced samples possess better strength, even after the severely conditioned freeze-thaw procedures.

**Figure 8:**
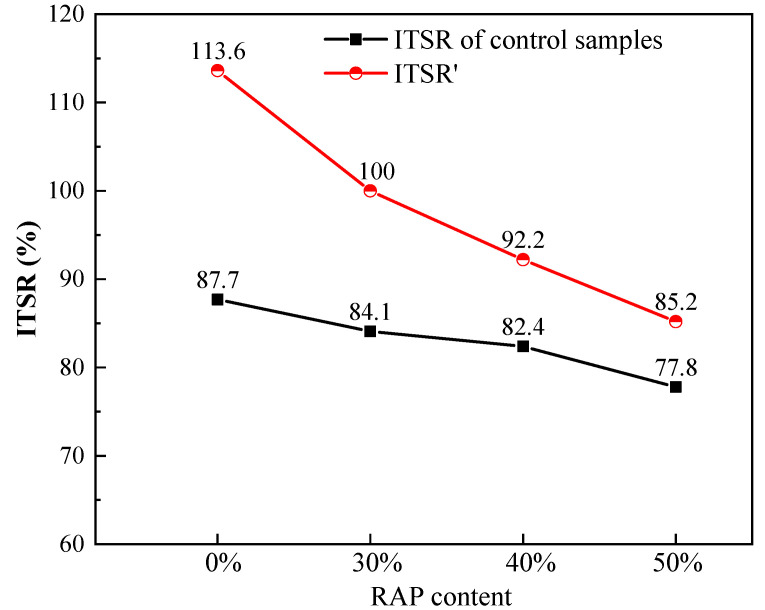
Comparisons of the indirect tensile strength ratio (ITSR) of control samples to the ITSR’.

Therefore, a new index of the ITSR’ was defined by the ITS of conditioned samples with BF divided by the ITS of unconditioned control samples, as shown in Equation (10). The comparisons of the ITSR of control samples to the ITSR’ were plotted in Figure 8, as they shared the same denominator as the benchmark for comparison.

(10) $${ITSR}^{\prime }=\frac{ITS\;of\;conditioned\;samples\;with\;BF}{ITS\;of\;unconditioned\;control\;samples}\times 100\%$$

It can be observed from Figure 8 that the ITSR’ showed a tremendous growth compared to the ITSR of control samples. The ITSR’ values increased by 25.9, 15.9, 9.8 and 7.4 percentage points for asphalt mixtures with 0%, 30%, 40% and 50% RAP, respectively. From this perspective, adding basalt fiber can enhance the moisture susceptibility of asphalt mixtures with RAP significantly. These findings are in accordance with the results in [42]. This reinforcement may be due to the spatial network structure formed by basalt fiber in the asphalt mixtures.

### 4.3. Effect of Basalt Fiber on Low-Temperature Performance of Asphalt Mixtures with RAP

**Figure 9:**
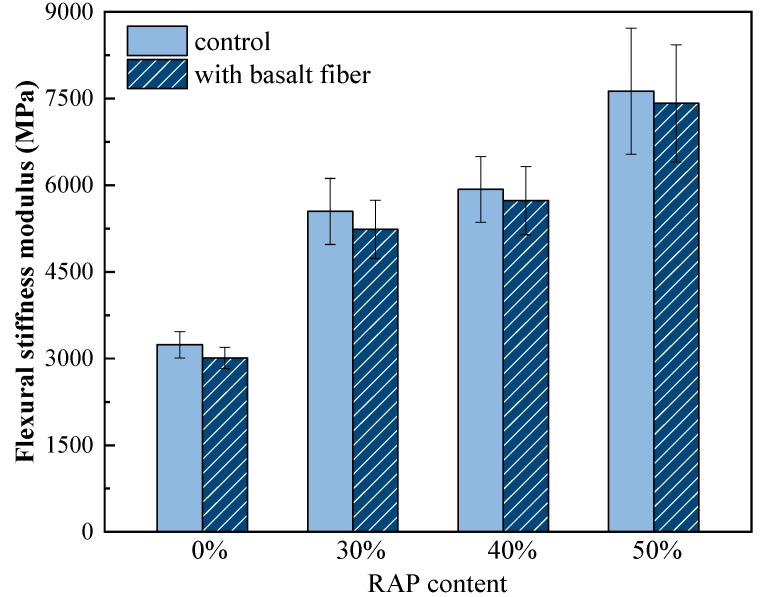
Flexural stiffness modulus of control and basalt fiber-reinforced asphalt mixtures

**Figure 10:**
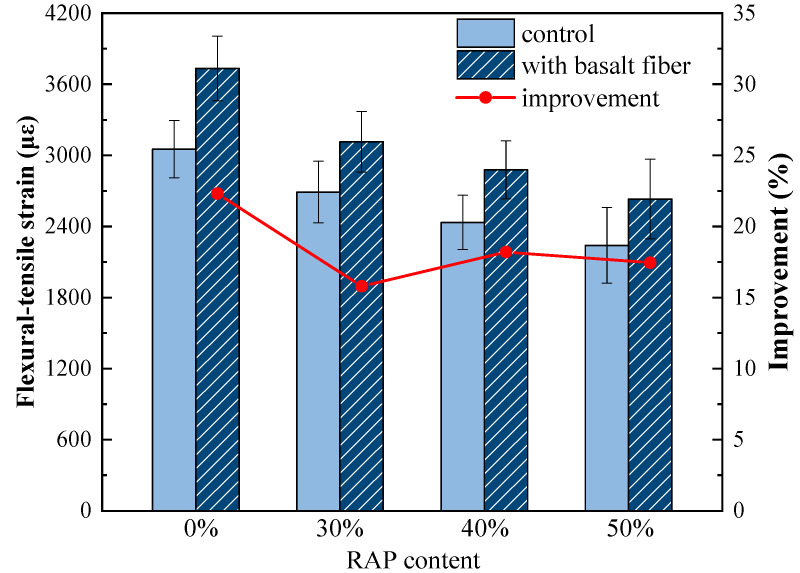
Flexural-tensile strain of control and basalt fiber-reinforced asphalt mixtures.

The flexural stiffness modulus and flexural-tensile strain results from the low-temperature bending beam test are shown in Figure 9 and Figure 10. It can be seen from Figure 9 that the flexural stiffness modulus of the control samples increased dramatically with an increasing RAP content. For instance, the stiffness modulus of asphalt mixtures increased by 71.3% when 30% RAP was added. Meanwhile, the stiffness modulus decreased to a certain extent by introducing basalt fiber to the samples with each RAP content. This means that basalt fiber can compensate for the stiffening impact caused by RAP and make the relevant asphalt mixtures more flexible at a low temperature, which is potentially beneficial to the low-temperature cracking resistance.

As shown in Figure 10, the flexural-tensile strain values of the control samples descended greatly. For instance, the flexural-tensile strain decreased from 3053 με to 2240 με when the RAP content rose from 0% to 50%, indicating the negative impact caused by RAP on the low-temperature cracking resistance of asphalt mixtures. With regard to the fiber-reinforced asphalt mixtures, the flexural-tensile strain values improved greatly for the samples with each RAP content, and these results are in line with the findings in [34]. The improvement ratio of flexural-tensile strain caused by basalt fiber was plotted as the red line in Figure 10. Compared to the control samples without basalt fiber, the flexural-tensile strain values increased by 22.3%, 15.8%, 18.2% and 17.5% for the fiber-reinforced asphalt mixtures with 0%, 30%, 40%, and 50% RAP, respectively. This means that adding basalt fiber does improve the low-temperature performance of asphalt mixtures with RAP. This may be due to the high fracture strength and superior elongation at the break of basalt fiber, which can bear and transit the stress and delay the fracture development [34].

### 4.4. Effect of Basalt Fiber on Cracking Resistance of Asphalt Mixtures with RAP

#### 4.4.1. SCB Fracture Test Results

**Figure 11:**
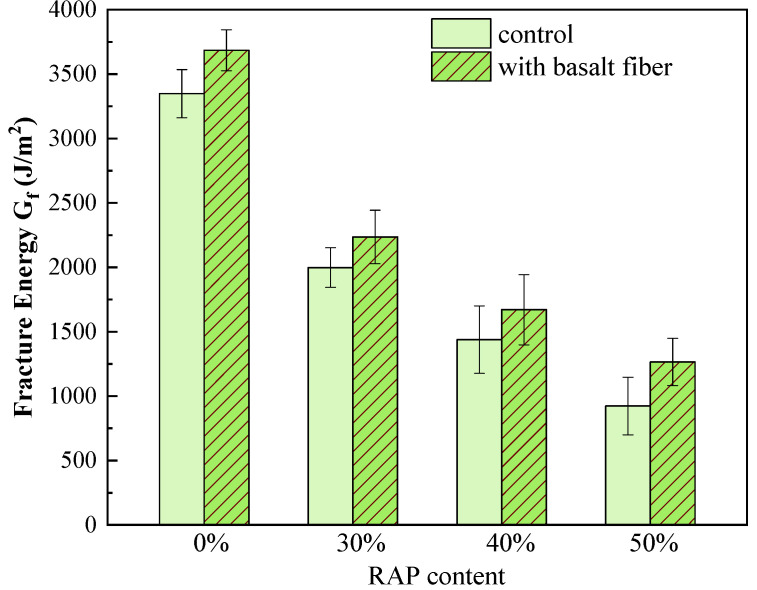
Fracture energy of control and basalt fiber-reinforced asphalt mixtures.

**Figure 12:**
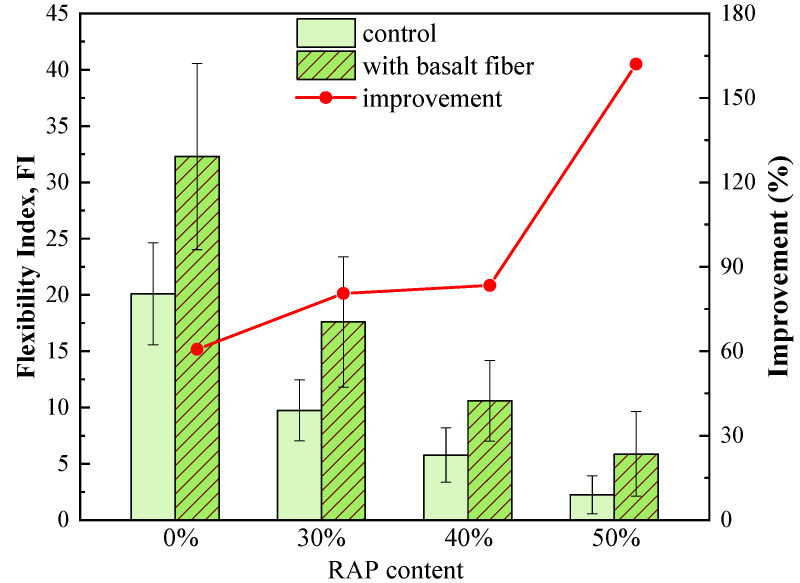
Flexibility index of control and basalt fiber-reinforced asphalt mixtures.

The fracture energy G_f_ and the flexibility index (FI) from the SCB fracture test are illustrated in Figure 11 and Figure 12. It can be seen from Figure 11 that the G_f_ values of the control samples dropped sharply with an increasing RAP content. For instance, the G_f_ of asphalt mixtures declined from 3348J/m^2^ with 0% RAP to 924 J/m^2^ with 50% RAP. This indicates that incorporating RAP into asphalt mixtures reduces its toughness and subsequently leads to inferior cracking resistance. In terms of the fiber-reinforced samples, the G_f_ values increased compared to the control ones. Specifically, the G_f_ increased by 10.1%, 11.9%, 16.1% and 37.0% for the mixtures with 0%, 30%, 40% and 50% RAP, respectively. The higher the RAP content, the greater the improvement in the fracture energy of asphalt mixtures caused by basalt fiber.

As shown in Figure 12, flexibility index (FI) values of the control samples also presented a sharp falling trend by increasing the RAP content, indicating a huge reduction in the cracking resistance of asphalt mixtures. However, FI values improved dramatically by introducing basalt fiber. The improvement ratio of FI caused by basalt fiber was plotted as the red line in Figure 12. Compared to the control sample, the FI values increased by 60.7%, 80.5%, 83.4% and 162.1% for the fiber-reinforced asphalt mixtures with 0%, 30%, 40%, and 50% RAP, respectively. The higher the RAP content, the greater the improvement ratio in the FI of asphalt mixtures. These findings are in line with the previous research [26]. One possible explanation for this result would be that the network structure of basalt fiber can reduce the stress concentration and delay crack propagation at an intermediate temperature. It is worth noting that the SCB fracture test is very sensitive to asphalt mixtures with RAP, as both G_f_ and FI presented a distinct decrease when RAP was used. However, the coefficient of variation (COV) is relatively high, especially for the FI values with the COV range of 22.5% to 74.8%. Furthermore, the COV of FI values increased with an increase in RAP content. 

#### 4.4.2. IDEAL Cracking Test Results

**Figure 13:**
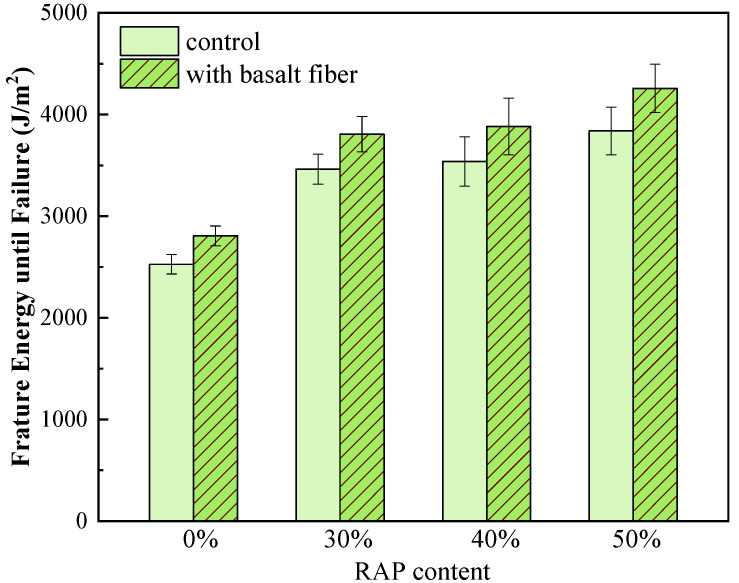
Fracture energy until failure of control and basalt fiber-reinforced asphalt mixtures.

**Figure 14:**
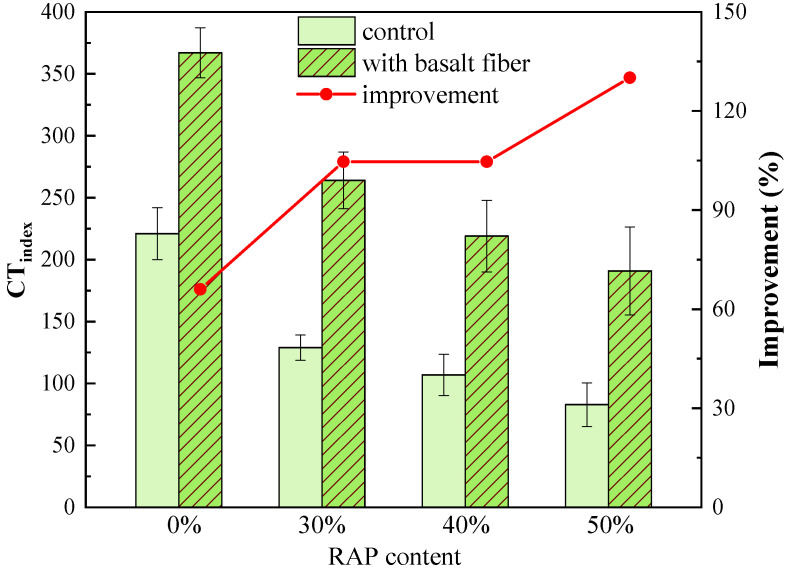
CT_index_ of control and basalt fiber-reinforced asphalt mixtures.

The fracture energy until failure and the CT_index_ from the IDEAL cracking test are shown in Figure 13 and Figure 14. It can be observed from Figure 13 that the fracture energy until failure enhanced greatly by increasing the RAP content for the control samples. This indicates that asphalt mixtures with RAP can bear higher fracture energy until the crack initiation. The reason for this could be that adding RAP material makes the mixtures stiffer and more brittle, and the load-bearing capacity of the mixtures dominates the impact on the fracture energy rather than the resultant strain [31]. Therefore, mixtures with RAP can bear higher stress results in enhanced fracture energy until failure. Besides, the fracture energy until failure of the fiber-reinforced mixtures further increased compared to those of the control samples with each RAP content. This result can be due to the strengthening effect caused by basalt fiber.

As shown in Figure 14, CT_index_ values of the control samples also exhibited a deep drop trend with an increasing RAP content. This infers that using RAP will contribute to the accelerated cracking propagation of asphalt mixtures. However, CT_index_ values also grew significantly by adding basalt fiber. The improvement ratio of CT_index_ caused by basalt fiber is shown as the red line in Figure 14. Compared to the control sample, the CT_index_ increased by 66.1%, 104.6%, 104.7% and 130.1% for the fiber-reinforced asphalt mixtures with 0%, 30%, 40%, and 50% RAP, respectively. The higher the RAP content, the greater the improvement ratio in the CT_index_ of asphalt mixtures. This indicates that basalt fiber can effectively slow down the cracking propagation rate, and subsequently enhance the cracking resistance of asphalt mixtures with RAP. Besides, it is clear that the CT_index_ is also rather sensitive to the impact caused by RAP on the cracking resistance of asphalt mixtures. Though it increased with an increasing RAP content, the COV of CT_index_ values was within the range of 5.5% to 21.2%, which was much smaller than those of FI values. Therefore, the CT_index_ results were chosen for further discussions in the next section.

### 4.5. Performance-Space Diagram Analysis

**Figure 15:**
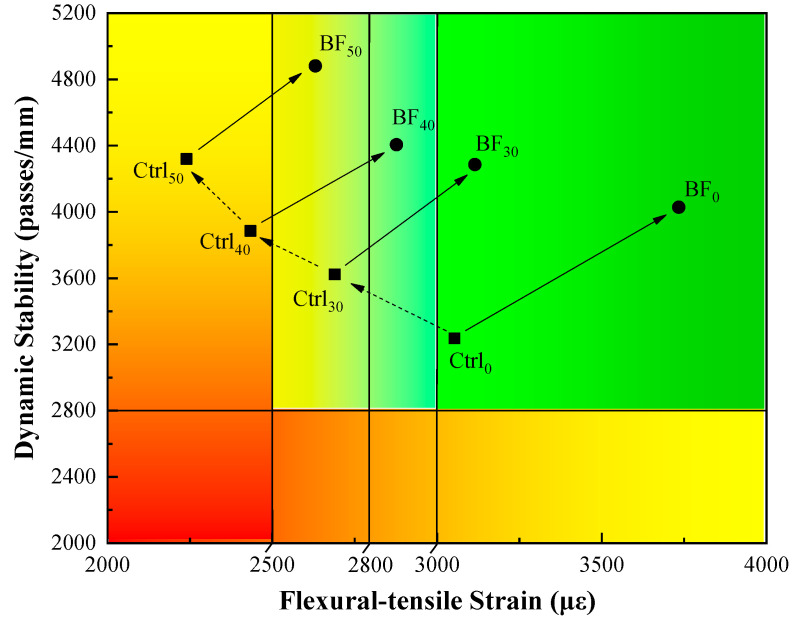
Dynamic stability-flexural-tensile strain (DS-FTS) performance-space diagram.

Buttlar [47] proposed the “Hamburg-DC (T) Performance-Space Diagram” to evaluate the high- and low-temperature performance of asphalt mixtures through a two-dimensional view. This diagram provides a visualized tool to determine the effect of additives and (or) alternative materials on the rutting and cracking behavior of asphalt mixtures simultaneously. Inspired by this diagram, the results from the wheel-tracking test and low-temperature bending beam test were chosen to represent the high- and low-temperature mixture performance in order to develop the so-called “dynamic stability-flexural-tensile strain (DS-FTS) performance-space diagram”. Specifically, dynamic stability (DS) and flexural-tensile strain (FTS) values were plotted on the Y-axis and X-axis, respectively, as shown in Figure 15. 

According to the Chinese standard JTG F40, the wheel-tracking test is unusually accompanied with three levels of DS thresholds based on climate regions (1800, 2400, and 2800 passes/mm for summer standard hot regions, summer very hot regions and summer extremely hot regions, respectively). Meanwhile, the low-temperature bending beam test also comes with three levels of FTS thresholds (2500, 2800, and 3000 με for winter standard and heavy cold regions, winter very cold regions and winter extremely cold regions, respectively). The summer extremely hot region and all the cold regions have been identified on this diagram. The control group samples were marked as “Ctrl” with the RAP content as the subscript, while the fiber-reinforced samples were marked as “BF”. Moreover, the effects of RAP and basalt fiber on the mixture performance shift were denoted by the dotted-line and solid-line arrows, respectively.

It can be seen from Figure 15 that the control samples with 0% RAP (marked as Ctrl_0_) presented qualified high- and low-temperature performance. The mixture performance moved to the upper-left section with an increasing RAP content. This indicates that the mixtures have superior rutting resistance, but risk high cracking potential. It is noteworthy that 30% RAP lead to a two-level degradation in FTS, which means that the low-temperature performance of mixtures shifted from the “winter extremely cold region” to the “winter standard and heavy cold regions”. Furthermore, both mixtures with 40% and 50% RAP failed in the FTS requirement and were recognized as unqualified mixtures. Meanwhile, it is clear that the mixture performance moved toward to the upper-right section by introducing basalt fiber to mixture with each RAP content. This means that both rutting and cracking resistance have been enhanced desirably. Specifically, basalt fiber resulted in a two-level bump in FTS for the mixtures with 30% and 40% RAP, and a one-level bump for the mixtures with 50% RAP. Therefore, compared to the control mixtures with 0% RAP, the mixtures with 30% RAP exhibited competitive low-temperature performance but better rutting resistance when basalt fiber was used. Moreover, due to the reinforcement caused by basalt fiber, the performance of unqualified mixtures with 50% RAP shifted into the same climate region as that of the control mixtures with 30% RAP. This infers that using basalt fiber can improve the performance of asphalt mixtures with RAP, or maintain the performance at a desirable level while increasing RAP content. 

**Figure 16:**
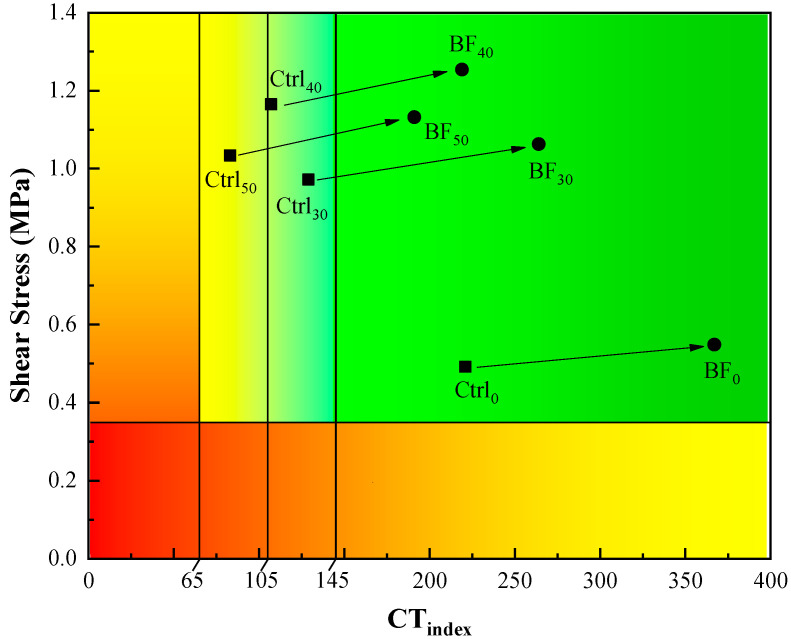
Shear stress (SS)-CT_index_ performance-space diagram.

In addition, the shear stress (SS) results from the uniaxial penetration test and CT_index_ results from the IDEAL cracking test were also plotted on the Y-axis and X-axis, respectively in order to develop the “SS-CT_index_ performance-space diagram”, as shown in Figure 16. Zhou [46] proposed three levels of CT_index_ thresholds based on mixture gradation type (65, 105, 145 for dense-graded, superpave, and Stone Matrix Asphalt (SMA) gradation, respectively), which were identified on this plot. It can be seen from Figure 16 that the mixture performance also shifted to the upper-left section with an increasing RAP content. However, the CT_index_ of the control mixtures with RAP still fell into the qualified zone. Even the CT_index_ of mixtures with 50% RAP meet the requirement for dense-graded gradation, while the mixtures with 30% and 40% RAP present comparable CT_index_ to that of superpave gradation. It is of no doubt that the mixture performance moved toward to the upper-right section again by adding basalt fiber, indicating that both rutting and cracking resistance have been enhanced for mixtures with each RAP content. Moreover, the CT_index_ of all the mixtures with basalt fiber met the requirement for SMA gradation. This means that using basalt fiber can slow down the crack propagation rate to a great extent. However, the CT_index_ thresholds proposed so far may not be universally applicable. More tests and corrections need to be conducted for both lab and filed mixtures.

## 5. Conclusions

This study has evaluated the effect of basalt fiber on the pavement performance of asphalt mixtures with high RAP contents by means of high- and low-temperature resistance, moisture susceptibility, and intermediate-temperature cracking resistance according to a series of laboratory tests. The performance-space diagram was plotted to analyze the performance changes caused by basalt fiber. Based on the analysis and discussion mentioned previously, the following conclusions can be drawn:

(1) Basalt fiber further enhances the high-temperature performance of asphalt mixtures by increasing both of the dynamic stability and shear stress, despite the stiffening effect caused by RAP. However, shear stress presents a declining trend when RAP content exceeds 40%, indicating a reduction in rutting resistance when excessive RAP material is used.
(2) Basalt fiber improves the indirect tensile strength (ITS) of conditioned and unconditioned samples. Based on the ratio of ITS of the conditioned fiber-reinforced samples when compared with the unconditioned control samples, superior moisture susceptibility of the fiber-reinforced mixtures could be determined, especially for the mixtures with RAP.
(3) Basalt fiber compensates for the stiffening impact caused by RAP at low temperature, subsequently resulting in better low-temperature cracking resistance of asphalt mixtures with RAP.
(4) Basalt fiber not only increases the fracture energy before crack initiation, but also slows down the cracking propagation rate at an intermediate temperature, meaning an overall improvement in intermediate-temperature cracking resistance of asphalt mixtures with RAP.
(5) Based on the “DS-FTS performance-space diagram”, basalt fiber improves both rutting and cracking resistance of asphalt mixtures with RAP simultaneously to a great extent. When basalt fiber is used, mixtures with 30% RAP exhibit comparable performance to control mixtures with 0% RAP, while unqualified mixtures with 50% RAP present a competitive performance to control mixtures with 30% RAP.
(6) Overall, adding basalt fiber can improve the performance of asphalt mixtures with RAP significantly, or increase the RAP content while maintaining the desired performance.

This study used an extensive range of laboratory tests to access the pavement performance of mixtures reinforced by basalt fiber. However, based on the recommendation from T/CHTS 10016-2019, only the basalt fiber with a length of 6 mm and a dosage of 0.3% were used. Different fiber lengths and dosage could be considered in future studies. Besides, a fatigue test and more low-temperature cracking tests such as DC (T) test (American standard ASTM D7313) could be performed to make a comprehensive investigation into the cracking resistance caused by basalt fiber. Furthermore, a life-cycle cost analysis is also a very interesting topic for future research.

## Figures and Tables

**Table 1:** Main physical and mechanical properties of basalt fiber.

Index	Unit	Value	Standard
Color	-	Golden brown	Visual inspection
Specific gravity	g/cm^3^	2.72	JT/T 776.1
Length	mm	6	JT/T 776.1
Diameter	μm	16	GB/T 7690.5
Fracture strength	MPa	2200	GB/T 20310
Elastic modulus	GPa	90	GB/T 20310
Thermostability (retained Fracture strength)	%	93	GB/T 7690.3
Elongation at break	%	2.7	JT/T 776.1
Oil absorption	%	52	JT/T 776.1
Water absorption	%	0.1	JT/T 776.1

**Table 2:** Properties of asphalt binder.

Index	Unit	Value	Standard
Penetration at 25 °C	0.1 mm	71	JTG E20 T0604
Penetration Index	-	0.5	JTG E20 T0604
Ductility at 5 °C	cm	48	JTG E20 T0605
Softening point	°C	64	JTG E20 T0606
Viscosity at 135 °C	Pa·s	1.8	JTG E20 T0625

**Table 3:** Binder content of the reclaimed asphalt pavement (RAP) and properties of the extracted binder.

Index	Unit	RAP_1_	RAP_2_	RAP_3_	RAP_4_	Average
Binder content	%	4.31	4.33	4.26	4.23	4.28
Penetration at 25 °C	0.1 mm	37.7	38.5	38.5	37.9	38.2
Softening point	°C	57.5	57.2	57.3	57.9	57.4
Viscosity at 135 °C	Pa·s	1.73	1.84	1.86	1.60	1.76

**Table 4:** Aging degree classification of the extracted binder.

Index	Neat Asphalt		SBS Modified Asphalt					
Viscosity (Pa·s)	η ≤ 1.6		η ≤ 1.6	1.6 < η ≤ 3			η > 3	
Penetration (0.1 mm)	P > 30	10 < P ≤ 30	P > 30	P > 30	20 < P ≤ 30	10 < P ≤ 20	20 < P ≤ 30	10 < P ≤ 20
Grade	I	II	I	II	III	IV	V	VI

**Table 5:** Gradations of asphalt mixtures with different RAP content.

Sieve Size (mm)	16	13.2	9.5	4.75	2.36	1.18	0.6	0.3	0.15	0.075
	Passing of Combined Aggregate (%)									
Upper limit	100.0	100.0	85.0	68.0	50.0	38.0	28.0	20.0	15.0	8.0
Lower limit	100.0	90.0	68.0	38.0	24.0	15.0	10.0	7.0	5.0	4.0
Mid-Range	100.0	95.0	76.5	53.0	37.0	26.5	19.0	13.5	10.0	6.0
0% RAP	100.0	98.3	80.4	51.6	34.1	20.9	14.4	9.7	7.8	6.7
30% RAP	100.0	96.9	80.6	50.2	35.4	24.0	16.7	12.4	9.6	7.8
40% RAP	100.0	96.5	81.0	50.3	35.2	24.3	16.9	12.6	9.6	7.7
50% RAP	100.0	96.1	81.3	50.4	34.3	24.3	16.7	12.7	9.5	7.4
